# Cardiac glycosides use and the risk of lung cancer: a nested case–control study

**DOI:** 10.1186/1471-2407-14-573

**Published:** 2014-08-08

**Authors:** Sébastien Couraud, Laurent Azoulay, Sophie Dell’Aniello, Samy Suissa

**Affiliations:** Centre for Clinical Epidemiology, Lady Davis Institute for Medical Research, Jewish General Hospital, Montreal, H3T 1E2 Quebec Canada; Department of Epidemiology, Biostatistics and Occupational Health, McGill University, Montreal, Quebec Canada; Pulmonology unit, Lyon Sud hospital, Hospices Civils de Lyon, Pierre Bénite, France; The faculty of medicine Lyon-Sud Charles Mérieux, Lyon 1 University, Oullins, France; Department of Oncology, McGill University, Montreal, Quebec Canada

**Keywords:** Lung cancer, Cardiac glycoside, Digoxin, Case–control study, Risk factor

## Abstract

**Background:**

Two studies have reported statistically significant associations between the use of cardiac glycosides (CGs) and an increased risk of lung cancer. However, these studies had a number of methodological limitations. Thus, the objective of this study was to assess this association in a large population-based cohort of patients.

**Methods:**

We used the United Kingdom Clinical Practice Research Datalink (CPRD) to identify a cohort of patients, at least 40 years of age, newly-diagnosed with heart failure, or supra-ventricular arrhythmia. A nested case–control analysis was conducted where each incident case of lung cancer identified during follow-up was randomly matched with up to 10 controls. Exposure to CGs was assessed in terms of ever use, cumulative duration of use and cumulative dose. Rate ratios (RRs) with 95% confidence intervals (CIs) were estimated using conditional logistic regression after adjusting for potential confounders.

**Results:**

A total of 129,002 patients were included, and followed for a mean (SD) of 4.7 (3.8) years. During follow-up, 1237 patients were newly-diagnosed with lung cancer. Overall, ever use of CGs was not associated with an increased risk of lung cancer when compared to never use (RR = 1.09, 95% CI: 0.94-1.26). In addition, no dose–response relationship was observed in terms of cumulative duration of use and cumulative dose with all RRs around the null value across quartile categories.

**Conclusion:**

The results of this large population-based study indicate that the use of CGs is not associated with an increased risk of lung cancer.

## Background

Cardiac glycosides (CGs) are natural steroids, derived from digitalis, that share a chemical structure with estrogens and are therefore considered phytoestrogens. The CG family includes digoxin, digitoxin and lanatoside C which remain important drugs in the treatment of atrial fibrillation (AF), some types of heart failure (HF), atrial flutter (AFl) and other supra-ventricular tachycardia (SVT) [1, 2].

Due to their ability to bind to estrogen receptors, [3] there has been interest in assessing whether the use of CGs is associated with the incidence of breast cancer. [4–9] Namely, two case–control studies found that the use of digoxin was associated with an increased risk of breast cancer (RR: 1.30, 95% CI: 1.14-1.48 and RR: 1.39, 95% CI: 1.32-1.46), respectively. [4, 5] There has also been interest on the effects of CGs on the incidence of lung cancer. Indeed, there are data supporting a role of female sexual hormones on lung cancer carcinogenesis, [10] which raises the hypothesis that the use of CGs may be associated with an increased risk of lung cancer. The main epidemiologic argument is the dramatic increase of non-small cell lung cancer in women over the last decades. [11] In addition, some observational studies found an association between lung cancer and some reproductive factors. [12–14] This biological rational is supported by the finding that estrogen receptors are frequently expressed in lung cancer tumors [15–17].

To date, only two only observational studies have investigated the link between the use of CGs and lung cancer incidence. [11, 12] In one study, the use of digitalis-related compounds was associated with a 65% increased risk of death from lung cancer. [11] In the other study, digitoxin users were found to have a significantly higher incidence of lung cancer compared to a matched control population (standardized incidence ratio: 1.35, 95% confidence interval [CI]: 1.04-1.74). [12] However, lung cancer was a secondary outcome in these studies, and the models were not adjusted for important potential confounders, such as smoking.

Given the limited data assessing the association between the use of CGs and the risk of lung cancer, we conducted a large population-based study to investigate whether the use of these drugs are associated with an increased risk of lung cancer in patients newly-diagnosed with HF, AF, AFl and/or SVT.

## Methods

### Data source

This study was conducted using the United Kingdom (UK) Clinical Practice Research Datalink (CPRD), formerly known as the General Practice Research Database. The CPRD is the world largest databank on primary care. Since its inception in 1987, it systematically records medical diagnoses and procedures, drug prescriptions issued by general practitioners, patient characteristics (such as body mass index [BMI]), and lifestyle factors (such as smoking and alcohol use). [13] Currently, the CPRD contains data on over 12 million patients registered with more than 650 participating general practices across the UK. Medical diagnoses and procedures are coded using the Read classification, and drugs are coded based on the UK Prescription Pricing Authority Dictionary. Cancer diagnoses, including lung cancer, in the CPRD have been shown to have a high validity [14].

The study protocol was approved by the Independent Scientific Advisory Committee of the CPRD and the Research Ethics Board of the Jewish General Hospital, Montreal, Quebec, Canada.

### Study population

Within the CPRD population, we identified all patients diagnosed for the first time with HF, AF, AFl and/or SVT, between January 1, 1988 and December 31, 2010, and followed until December 31, 2012. Cohort entry was defined as the date of any of the previously considered diagnoses, whichever appeared first in the patient’s medical record. The cohort was then restricted to patients at least 40 years of age at cohort entry, and those with at least two years of ‘up-to-standard’ medical history in the general practice prior to cohort entry. In order to identify new users of CGs during follow-up, we excluded all patients who previously received these drugs at any time prior to cohort entry. Finally, we excluded all patients previously diagnosed with any cancer (excluding non-melanoma skin cancer) at any time prior to cohort entry to ensure the identification of incident cases of lung cancer during follow-up, and to avoid the inclusion of patients with metastatic disease to the lung from other cancer sites. Patients meeting the study inclusion criteria were then followed until a first-ever diagnosis of lung cancer, death from any cause, end of registration with the general practice, or end of the study period (December 31, 2012), whichever came first.

### Case–control selection

Within the cohort defined above, we conducted a nested case–control analysis, which produces odds ratios that are unbiased estimators of rate ratios (RRs) (i.e. no need for the rare disease assumption) [15].

Cases consisted of all those newly-diagnosed with lung cancer during follow-up. Up to 10 controls were randomly selected from the case’s risk set (i.e. subset of the cohort still at risk of experiencing the outcome at the time of the case’s event date), after matching on year of birth (±1 year), sex, cohort entry date (±1 year), and duration of follow-up. The date of each case’s lung cancer diagnosis defined the index date, which was also assigned to the matched controls. All controls were alive, not previously diagnosed with lung cancer, and registered with their general practice when matched to a given case. All analyses were restricted to cases and matched controls with at least one year of follow-up in the risk set, which was necessary for latency considerations.

### Exposure to cardiac glycosides

We obtained all prescriptions for CGs received between cohort entry and index date. We excluded exposures initiated in the year immediately prior to index date in order to take into account a latency time window (lag time), and to minimize reverse causality, where initiation or termination of a treatment may have been influenced by early signs or symptoms of lung cancer.

For the primary analysis, exposure to CGs was defined as receiving at least one prescription of digoxin, lanatoside C, digitoxin, or digitalis, between cohort entry and the year prior to index date. For the secondary analysis, we assessed whether there was a dose–response relationship in terms of CG cumulative duration of use and cumulative dose. Therefore, for patients deemed to have ever used CGs, we calculated their cumulative duration of use, defined as the sum of the specified durations of all CGs prescription received between cohort entry and index date. Cumulative dose was computed by multiplying the daily dose of each CG prescription by its specified duration of use and then summing the total quantities received between cohort entry and index date. Since CGs include four different drugs, we used the “defined daily dose” (DDD) equivalence to convert digitalis, digitoxin and lanatoside C in digoxin equivalents doses (the most commonly used CG). Thus, 250 micrograms of digoxin was equivalent to 0.1 milligrams of digitoxin, to 100 milligrams of digitalis, and to 1 milligrams of lanatoside C. Cumulative duration of use and cumulative dose were classified in quartile categories based on the distribution of use in the controls.

### Statistical analysis

Descriptive statistics were used to describe the characteristics of the cohort, cases and matched controls. We used conditional logistic regression to estimate RRs and 95% CIs. In the primary analysis, we assessed whether the use of CGs was associated with an increased risk of lung cancer. In the second analysis, we determined whether there was a dose–response relationship in terms of cumulative duration of use and cumulative dose.

In addition to the matching variables (age, sex, year of cohort entry, and duration of follow-up) on which the logistic regression was conditioned, the models were adjusted for the following potential confounders measured at least one year prior to index date: smoking status, BMI (<18.50 kg/m^2^, 18.50-24.99 kg/m^2^, 25.00-29.99 kg/m^2^, ≥ 30.00 kg/m^2^), indication of CG use (HF, AF, AFl and/or SVT), excessive alcohol use, history of tobacco-related conditions (chronic obstructive pulmonary disease, ischemic heart disease, and vascular diseases), history of lung diseases (pneumonia, tuberculosis, and history of chronic lung disease), and factors associated with sexual hormonal disorders (hypothalamic, pituitary, testis, ovarian and adrenal gland disorders as well as virilism, hormonal infertility, secondary and primary hormonal deficiency). We also adjusted for drugs potentially associated with lung cancer incidence (also measured at least one year prior to index date), which consisted of statins, aspirin, oral anticoagulants and antiplatelets, non-steroidal anti-inflammatory drugs, anti-hypertensives (diuretics including spironolactone, calcium-channel blockers, angiotensin receptor blockers, angiotensin converting enzyme inhibitors, and beta-blockers), oral bisphosphonates, anti-diabetic drugs (metformin, sulfonylureas, insulins, thiazolidinediones, and other anti-diabetic agents), and amiodarone (which is rather implicated in chronic interstitial pneumonia and commonly prescribed in supraventricular arrhythmia). Variables with missing information were coded with an ‘unknown’ category.

### Sensitivity and subgroup analyses

We conducted two sensitivity analyses to assess the robustness of the results. In the first, we varied the lag period prior to index date from one year to six months and two years. The shorter six-month lag period was considered to account for lung cancer’s usual rapid growth. In the second analysis, we additionally adjusted the models for the use of hormone replacement therapy and estrogen-based contraceptives among the female subgroup of cases and matched controls. We also conducted a subgroup analysis, where we assessed whether smoking status, which is the leading risk factor for lung cancer, was an effect modifier of the association between the use of CGs and lung cancer. For this analysis, effect modification was assessed by including interaction terms in the model between CG use and smoking. All analyses were conducted with SAS version 9.3 (SAS Institute, Cary, NC).

## Results

**Figure 1:**
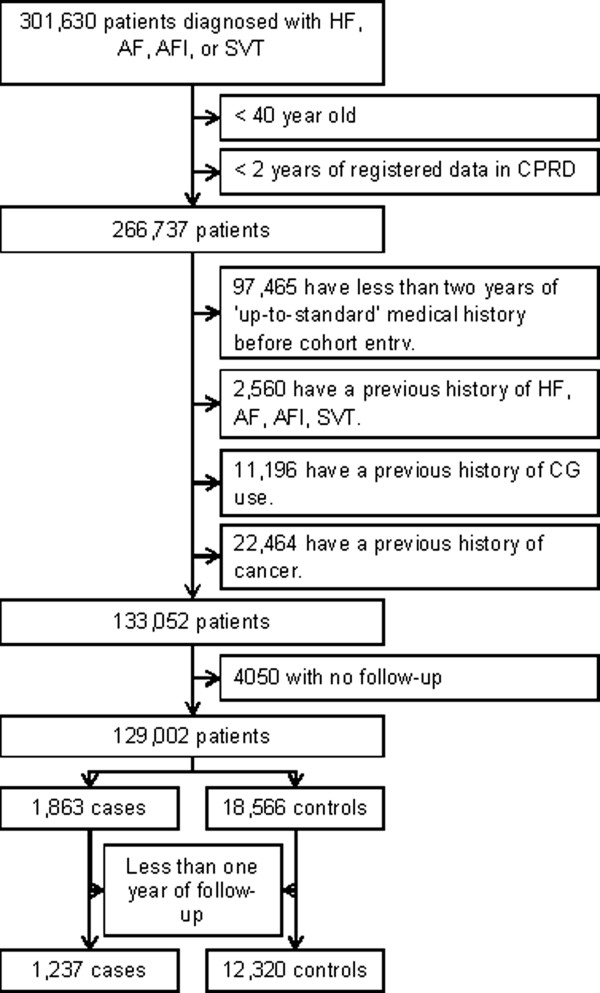
**Flow chart of the cohort.** CPRD – Clinical Practice Research Datalink; HF – Heart Failure; AF - Atrial fibrillation; AFl – Atrial Flutter; SVT – Supra Ventricular Tachycardia; CGs – Cardiac Glycosides.

A total of 129,002 patients met the study inclusion criteria (Figure 1). The cohort comprised 65,369 men (50.7%) and the mean (standard deviation [SD]) age at cohort entry was 73.9 (11.5) years. Overall, 69,865 (54.2%) patients were diagnosed with AF, 55,240 (42.8%) with HF, and 6605 (5.1%) with AFl or SVT. Patients were followed for a mean (SD) of 4.7 (3.8) years, generating 610,954 person-years of follow-up. A total of 1237 patients were newly-diagnosed with lung cancer during follow-up, generating an incidence rate of 202/100,000 (95% CI: 191–214) persons per year.

The characteristics of the cases and matched controls are shown in Table 1. As expected, compared to controls, lung cancer cases were more likely to have been smokers, had a higher prevalence of COPD, history of pneumonia, and chronic lung diseases.

**Table 1:** **Characteristics of lung cancer cases and matched controls**

Characteristic	Cases	Controls
	n = 1237	n = 12,320
Age^a^, mean (SD)	77.3 (8.1)	76.8 (8.0)
<50 years, n (%)	2 (0.2)	20 (0.2)
50-70 years, n (%)	248 (20.0)	2695 (21.9)
>71 years, n (%)	987 (79.8)	9605 (78.0)
Male^a^, n (%)	818 (66.1)	8137 (66.0)
Follow-up time, years^a^; Mean (SD)	4.9 (3.2)	4.9 (3.2)
Smoking, n (%)		
Never	134 (10.8)	4780 (38.8)
Ever^b^	1031 (83.3)	6531 (53.0)
Unknown	72 (5.8)	1009 (8.2)
Excessive alcohol use, n (%)	143 (11.6)	970 (7.9)
Body mass index, n (%)		
<18.5 kg/m^2^	29 (2.3)	172 (1.4)
18.5 to 24.9 kg/m^2^	365 (29.5)	3095 (25.1)
25.0 to 29.9 kg/m^2^	390 (31.5)	4150 (33.7)
≥ 30.0 kg/m^2^	241 (19.5)	2782 (22.6)
Unknown	212 (17.1)	2121 (17.2)
Cohort entry indication^c^, n (%)		
Chronic heart Failure	612 (49.5)	4782 (38.8)
Atrial fibrillation	561 (45.4)	6873 (55.8)
Atrial flutter or supra ventricular tachycardia	64 (5.2)	665 (5.4)
Comorbidities, n (%)		
Chronic obstructive pulmonary disease	428 (34.6)	1658 (13.5)
Heart and vascular diseases	697 (56.3)	6042 (49.0)
Pneumonia	740 (59.8)	5716 (46.4)
Tuberculosis	22 (1.8)	224 (1.8)
Other chronic lung diseases	333 (26.9)	2194 (17.8)
Sexual hormone disorders	7 (0.6)	84 (0.7)
Concomitant drugs, n (%)		
Amiodarone	77 (6.2)	825 (6.7)
Non-steroid anti-inflammatory drugs	186 (15.0)	1954 (15.9)
Anti-hypertensives	1093 (88.4)	10701 (86.9)
Oral anticoagulants and antiplatelets	443 (35.8)	4941 (40.1)
Aspirin	578 (46.7)	5857 (47.5)
Statins	532 (43.0)	5240 (42.5)
Oral estrogen contraceptives and hormone replacement therapy^d^	17 (4.1)	114 (2.7)
Anti-diabetic agents	143 (11.6)	1453 (11.8)

^a^Matching variables along with year of cohort entry.

^b^Includes current and former smokers.

^c^Defined as the first ever recorded diagnosis.

^d^Among women only.

The results of the primary and secondary analyses are shown in Table 2. Overall, ever use of CGs was not associated with an increased risk of lung cancer when compared to never use (RR = 1.09, 95% CI: 0.94-1.26). In addition, no dose–response relationship was observed in terms of cumulative duration of use and cumulative dose with all RRs around the null value across the quartile categories. A total of 89 cases and 972 matched controls and 16 cases and 207 matched controls used CGs for more than 5 and 10 years. The use of CGs for at least 5 and 10 years was not associated with an increased risk of lung cancer (RR: 1.04, 95% CI: 0.78-1.39 and RR: 0.79, 95% CI: 0.45-1.39, respectively). Only two controls were exposed to digitoxin and lanatoside, while all other cases and controls were exposed to digoxin only.

**Table 2:** **Crude and adjusted rate ratios for the association between the use of cardiac glycosides and lung cancer incidence**

Exposure to cardiac glycosides	Cases	Controls	Crude RR	Adjusted RR (95% CI) ^a^
	(n = 1237)	(n = 12,320)		
**Overall**				
No use, n (%)	860 (69.5)	8528 (69.2)	1.00	1.00 (Reference)
Ever use, n (%)	377 (30.5)	3792 (30.8)	0.98	1.09 (0.94 - 1.26)
**Cumulative duration of use**				
<14 months, n (%)	102 (8.2)	962 (7.8)	1.04	1.14 (0.91 - 1.45)
14 - 32 months, n (%)	102 (8.2)	931 (7.6)	1.10	1.21 (0.96 - 1.54)
32 - 60 months, n (%)	84 (6.8)	927 (7.5)	0.89	0.97 (0.75 - 1.25)
> 60 months, n (%)	89 (7.2)	972 (7.9)	0.89	0.99 (0.76 - 1.29)
**Cumulative dose (in digoxin-equivalents)**				
< 60 mg, n (%)	121 (9.8)	1109 (9.0)	1.08	1.17 (0.94 - 1.45)
60 - 120 mg, n (%)	85 (6.9)	746 (6.1)	1.14	1.24 (0.96 - 1.59)
120 - 240 mg, n (%)	88 (7.1)	880 (7.1)	0.99	1.08 (0.84 - 1.39)
>240 mg, n (%)	83 (6.7)	1057 (8.6)	0.76	0.85 (0.65 - 1.11)

^a^Adjusted on smoking status BMI indication of CG use excessive alcohol use history of tobacco-related conditions history of lung diseases factors associated with sexual hormonal disorders drugs potentially associated with lung cancer (statins aspirin oral anticoagulants and antiplatelets non-steroidal anti-inflammatory drugs anti-hypertensives oral bisphosphonates anti-diabetic drugs) and amiodarone.

*RR* Rate ratio, *CGs* Cardiac Glycosides.

In sensitivity analyses, varying the lag period to six month and 2 years produced results consistent with those of the primary analysis (RR: 1.08, 95% CI: 0.94-1.24; and RR: 1.02, 95% CI: 0.86-1.20, respectively). In the female subgroup, further adjustment for hormone replacement therapy and estrogen contraceptives did not materially change the results (Table 3). Finally, smoking status was not an effect modifier of the association between the use of CGs and lung cancer (see Table 4).

**Table 3:** **Crude and adjusted rate ratios for the association between the use of cardiac glycosides and lung cancer by varying the lag period to 6 months and 2 years and by additionally adjusting for sexual hormone intake in women**

***Sensitivity analysis***	Cases	Controls	Crude RR	Adjusted RR (95% CI) ^a^
**6-month lag period**	**1423**	**14,166**		
No use, n (%)	997 (70.1)	9843 (69.5)	1.00	1.00 (Reference)
Ever use, n (%)	426 (29.9)	4323 (30.5)	0.97	1.08 (0.94 - 1.24)
**2-year lag period**	**990**	**9850**		
No use, n (%)	703 (71.0)	6815 (69.2)	1.00	1.00 (Reference)
Ever use, n (%)	287 (29.0)	3035 (30.8)	0.91	1.02 (0.86 - 1.20)
**Additionally adjusting for HRT/OC use in women**	**419**	**4183**		
No use, n (%)	295 (70.4)	2828 (67.6)	1.00	1.00 (Reference)
Ever use, n (%)	124 (29.6)	1355 (32.4)	0.88	0.97 (0.74 - 1.27)

^a^Adjusted on smoking status BMI indication of CG use excessive alcohol use history of tobacco-related conditions history of lung diseases factors associated with sexual hormonal disorders drugs potentially associated with lung cancer (statins aspirin oral anticoagulants and antiplatelets non-steroidal anti-inflammatory drugs anti-hypertensives oral bisphosphonates anti-diabetic drugs) and amiodarone.

*RR* Rate ratio, *HRT* Hormone replacement therapy, *OC* Oral contraceptive).

**Table 4:** **Effect modification by smoking status on the association between cardiac glycosides and lung cancer incidence**

Smoking status	Cases	Controls	Adjusted RR (95% CI) ^a^	P value for interaction
**Never**	**134**	**4780**		
No use, n (%)	92	3270	1.00 (Reference)	0.37
Ever use, n (%)	42	1510	1.08 (0.74-1.58)	
**Ever**	**1031**	**6531**		
No use, n (%)	722	4568	1.00 (Reference)	
Ever use, n (%)	309	1963	1.06 (0.90-1.24)	
**Unknown**	**72**	**1009**		
No use, n (%)	46	690	1.00 (Reference)	
Ever use, n (%)	26	319	1.55 (0.93-2.58)	

^a^Adjusted on BMI indication of CG use excessive alcohol use history of tobacco-related conditions history of lung diseases factors associated with sexual hormonal disorders drugs potentially associated with lung cancer (statins aspirin oral anticoagulants and antiplatelets non-steroidal anti-inflammatory drugs anti-hypertensives oral bisphosphonates anti-diabetic drugs) and amiodarone.

*RR* Rate Ratio.

## Discussion

The results of this large population-based study indicate that the use of CGs and is not associated with an increased risk of lung cancer in patients newly-diagnosed with HF, AF, AFl or SVT. In addition, there was no evidence of a dose- or duration-response relationship. Overall, the results remained robust in sensitivity analyses.

While our findings suggest no association between the use of CGs and lung cancer, two previous studies have reported increased risks. [11, 12] In the first study, the authors calculated standardized mortality rates, using computerized pharmacy records of 143,574 patients from 1969 to 1973, which included 2,466 CG users. [11] The authors reported significant associations between 215 drugs and 56 cancer sites. Among these drugs, the use of CGs were associated with an increased risk for all cancers, including lung cancer (SMR = 1.23 and 1.65 respectively, both p < .002, no 95% CIs were provided). In the second study which used the Norwegian Cancer Registry, users of CGs were found to have an increased risk of lung cancer, when compared to the general population (standardized incidence ratio: 1.35, 95% CI: 1.04-1.74). [12] However, both of these studies had important methodological limitations, such as lack of adjustment for potentially important confounders, including smoking, alcohol use, and comorbidity. [11, 12] In contrast, our analyses were adjusted for these variables, and residual confounding was further minimized by selecting a cohort of patients with indications associated with the use of CGs.

The lack of an association observed in our study is supported by biological evidence. Estrogen receptors are found on normal lung tissue samples, [16] and thus, since lung is usually modulated by sex hormone, it can be hypothesized that phytoestrogens would not specifically induce cells or tissue damage (more than sex hormones themselves). It is possible that sex hormones may act as an oncogenic trigger in a small subset of patients, possibly those predisposed to hormone-related cancers and who carry some particular polymorphisms in estrogen metabolism-related genes, as was previously suggested. [17] This hypothesis may also explain conflicting results regarding lung cancer risk and female reproductive factors. [10] Additional studies are needed to identify this subset of patients.

Moreover, recent studies have revealed potent anti-cancer activity of CGs *in vitro*, and some derivatives of CGs are currently being investigated for cancer therapies in clinical trials. [18, 19] CGs may act as inhibitors of hypoxia-induced factors and inducers of immunogenic cell death, possibly through the MAP Kinase pathway. However, only a few clinical studies have assessed the effect of CGs on oncogenesis, with heterogeneous findings. Therefore, the potential of CGs as anticancer drugs remains to be fully evaluated [20].

Our study has a number of strengths. First, we assembled a cohort among patients newly-diagnosed with HF, AF, AFl, or SVT. This was to minimize confounding by indication, which was a limitation of the previous studies. [11, 12] Second, we matched controls to cases on year of cohort entry, to minimize time trends in CG use and lung cancer incidence in the 25-year study period. Indeed, in UK as in most countries, CGs moved further down the management pathway of HF and AF. [21, 22] Third, the models were adjusted for smoking status, which is the major risk-factor for lung cancer which its absence was a limitation in the previous studies on this subject. [11, 12] However, despite the availability of smoking status in the CPRD, it was missing for 5.8% of cases and 8.2% of controls. However, to minimize any residual confounding, the models were additionally adjusted for smoking-related diseases (COPD, heart and vascular diseases).

Our study also has some limitations. First, drug information in the CPRD represents prescriptions written by general practitioners. As such, it is unknown whether prescriptions were actually filled at the pharmacy and whether patients fully complied with the treatment regimen. However, difference in compliance in not thought to be differentially distributed among cases and controls and should not have biased the results. Second, a limitation of the CPRD is the lack of information on certain lung cancer risk factors, such as occupational exposures to carcinogens, exposure to second-hand smoking, socio-economic status, and family history of lung cancer. [10, 23] For women, additional reproductive factors such as age at menopause or duration of sex life were not taken into account. However, while these factors may be weakly to moderately associated with lung cancer incidence, we do not believe they are necessarily associated with the use of CGs, thus unlikely to strongly confound the association.

## Conclusion

In summary, the results of this large population-based study indicate that the use of CGs is not associated with an increased risk of lung cancer. These findings should provide reassurance to physicians and patients using these agents.

## Consent

All data were anonymized for research purposes and thus did not require patient informed consent.

## References

[1] McMurray JJ, Adamopoulos S, Anker SD, Auricchio A, Böhm M, Dickstein K, Falk V, Filippatos G, Fonseca C, Gomez-Sanchez MA, Jaarsma T, Køber L, Lip GY, Maggioni AP, Parkhomenko A, Pieske BM, Popescu BA, Ronnevik PK, Rutten FH, Schwitter J, Seferovic P, Stepinska J, Trindade PT, Voors AA, Zannnad F, Zeiher A (2012). ESC Guidelines for the diagnosis and treatment of acute and chronic heart failure 2012: The Task Force for the Diagnosis and Treatment of Acute and Chronic Heart Failure 2012 of the European Society of Cardiology. Developed in collaboration with the Heart Failure Association (HFA) of the ESC. Eur Heart J.

[2] Camm AJ, Lip GYH, De Caterina R, Savelieva I, Atar D, Hohnloser SH, Hindricks G, Kirchhof P, Bax JJ, Baumgartner H, Ceconi C, Dean V, Deaton C, Fagard R, Funck-Brentano C, Hasdai D, Hoes A, Kirchhof P, Knuuti J, Kolh P, McDonagh T, Moulin C, Popescu BA, Reiner Z, Sechtem U, Sirnes PA, Tendera M, Torbicki A, Authors/Task Force Members (2012). 2012 focused update of the ESC Guidelines for the management of atrial fibrillation: An update of the 2010 ESC Guidelines for the management of atrial fibrillation * Developed with the special contribution of the European Heart Rhythm Association. Eur Heart J.

[3] Cove DH, Barker GA (1979). Digoxin and hormone receptors. Lancet.

[4] Biggar RJ, Wohlfahrt J, Oudin A, Hjuler T, Melbye M (2011). Digoxin use and the risk of breast cancer in women. J Clin Oncol.

[5] Ahern TP, Lash TL, Sørensen HT, Pedersen L (2008). Digoxin treatment is associated with an increased incidence of breast cancer: a population-based case–control study. Breast Cancer Res.

[6] Ewertz M, Holmberg L, Tretli S, Pedersen BV, Kristensen A (2001). Risk factors for male breast cancer–a case–control study from Scandinavia. Acta Oncol.

[7] Lenfant-Pejovic MH, Mlika-Cabanne N, Bouchardy C, Auquier A (1990). Risk factors for male breast cancer: a Franco-Swiss case–control study. Int J Cancer.

[8] Hartz AJ, He T (2013). Cohort study of risk factors for breast cancer in post menopausal women. Epidemiol Health.

[9] Ahern TP, Tamimi RM, Rosner BA, Hankinson SE (2014). Digoxin use and risk of invasive breast cancer: evidence from the Nurses’ Health Study and meta-analysis. Breast Cancer Res Treat.

[10] Couraud S, Zalcman G, Milleron B, Morin F, Souquet P-J (2012). Lung cancer in never smokers–a review. Eur J Cancer.

[11] Selby JV, Friedman GD, Fireman BH (1989). Screening prescription drugs for possible carcinogenicity: eleven to fifteen years of follow-up. Cancer Res.

[12] Haux J, Klepp O, Spigset O, Tretli S (2001). Digitoxin medication and cancer; case control and internal dose–response studies. BMC Cancer.

[13] Walley T, Mantgani A (1997). The UK general practice research database. Lancet.

[14] Dregan A, Moller H, Murray-Thomas T, Gulliford MC (2012). Validity of cancer diagnosis in a primary care database compared with linked cancer registrations in England. Population-based cohort study. Cancer Epidemiol.

[15] Breslow NE (1996). Statistics in epidemiology: the case–control study. J Am Stat Assoc.

[16] Omoto Y, Kobayashi Y, Nishida K, Tsuchiya E, Eguchi H, Nakagawa K, Ishikawa Y, Yamori T, Iwase H, Fujii Y, Warner M, Gustafsson JA, Hayashi SI (2001). Expression, function, and clinical implications of the estrogen receptor beta in human lung cancers. Biochem Biophys Res Commun.

[17] Cote ML, Yoo W, Wenzlaff AS, Prysak GM, Santer SK, Claeys GB, Van Dyke AL, Land SJ, Schwartz AG (2009). Tobacco and estrogen metabolic polymorphisms and risk of non-small cell lung cancer in women. Carcinogenesis.

[18] Kepp O, Menger L, Vacchelli E, Adjemian S, Martins I, Ma Y, Sukkurwala AQ, Michaud M, Galluzzi L, Zitvogel L, Kroemer G (2012). Anticancer activity of cardiac glycosides: At the frontier between cell-autonomous and immunological effects. Oncoimmunology.

[19] Elbaz HA, Stueckle TA, Tse W, Rojanasakul Y, Dinu CZ (2012). Digitoxin and its analogs as novel cancer therapeutics. Exp Hematol Oncol.

[20] Menger L, Vacchelli E, Kepp O, Eggermont A, Tartour E, Zitvogel L, Kroemer G, Galluzzi L (2013). Trial watch: Cardiac glycosides and cancer therapy. Oncoimmunology.

[21] Smith NL, Chan JD, Rea TD, Wiggins KL, Gottdiener JS, Lumley T, Psaty BM (2004). Time trends in the use of beta-blockers and other pharmacotherapies in older adults with congestive heart failure. Am Heart J.

[22] Pilote L, Eisenberg MJ, Essebag V, Tu JV, Humphries KH, Leung Yinko SSL, Behlouli H, Guo H, Jackevicius CA (2013). Temporal trends in medication Use and outcomes in atrial fibrillation. Can J Cardiol.

[23] Alberg AJ (2013). Epidemiology of lung CancerDiagnosis and management of lung cancer, 3rd ed: american college of chest physicians evidence-based clinical practice guidelines. Chest J.

[CR24] The pre-publication history for this paper can be accessed here:http://www.biomedcentral.com/1471-2407/14/573/prepub

